# Automatic point cloud registration algorithm based on the feature histogram of local surface

**DOI:** 10.1371/journal.pone.0238802

**Published:** 2020-09-11

**Authors:** Jun Lu, Zhuo Wang, Bowen Hua, Kun Chen

**Affiliations:** 1 College of Automation, Harbin Engineering University, Harbin, China; 2 Key laboratory of Intelligent Technology and Application of Marine Equipment, Harbin Engineering University, Ministry of Education, Harbin, China; University of Bradford, UNITED KINGDOM

## Abstract

In this paper, we present an efficient algorithm for point cloud registration in presence of low overlap rate and high noise. The proposed registration method mainly includes four parts: the loop voxel filtering, the curvature-based key point selection, the robust geometric descriptor, and the determining and optimization of correspondences based on key point spatial relationship. The loop voxel filtering filters point clouds to a specified resolution. We propose a key point selection algorithm which has a better anti-noise and fast ability. The feature descriptor of key points is highly exclusive which is based on the geometric relationship between the neighborhood points and the center of gravity of the neighborhood. The correspondences in the pair of two point clouds are determined according to the combined features of key points. Finally, the singular value decomposition and ICP algorithm are applied to align two point clouds. The proposed registration method can accurately and quickly register point clouds of different resolutions in noisy situations. We validate our proposal by presenting a quantitative experimental comparison with state-of-the-art methods. Experimental results show that the proposed point cloud registration algorithm has faster calculation speed, higher registration accuracy, and better anti-noise performance.

## 1. Introduction

With the development of novel sensing technologies, such as Kinect, 3D LiDAR [1, 2] and terrestrial laser scanners (TLS), 3D point cloud becomes more convenient to acquire. And those technologies have been used widespreadly in the fields of 3D reconstruction, archaeology, medical image analysis etc. Point cloud processing has become a research hotspot. To reconstruct a complete 3D model, it is necessary to obtain the point cloud from different viewpoints. But each point cloud is in different coordinate systems. Therefore, point clouds of multi-view in different coordinate systems should be transformed to one coordinate system. This process is called point cloud registration. Point cloud registration is a key step in point cloud processing and has the profound value in computer vision, computer graphics, robotics and so on.

According to the initial conditions and accuracy, point cloud registration can be divided into coarse registration and fine registration. The coarse registration can quickly estimate a rough transformation matrix without strict requirements of initial spatial positions of point clouds. The fine registration can obtain a good result of registration. There are numerous algorithms for point cloud registration proposed by scholars. Of these algorithms, the Iterative Closest Point (ICP) algorithm is an important registration method for fine registration [3]. The ICP algorithm proposed by Besl et al. can obtain the best transformation matrix according to correspondences iteratively. However, the ICP algorithm also has some shortcomings, such as high requirements for initial positions of point clouds. Chen et al. presented a new approach which works on range data directly and aligns successive scans with enough overlapping area to get an accurate transformation between scans [4].

Ji et al. proposed a hybrid algorithm which integrated the GA algorithm and the ICP algorithm [5]. In the literature [6], Zhu et al. deployed an improved Iterative Closest Point (ICP) algorithm in which an adaptive threshold was introduced to accelerate iterative convergence. Meng combined kd-tree and extrapolation to improve the speed and accuracy of the ICP algorithm [7]. In order to improve the accuracy of point cloud registration and the convergence speed of registration, Liu et al. took point pairs with smaller Euclidean distances as the points to be registered, and designed the depth measurement error model and weight function [8]. Agamennoni et al. presented a point cloud registration method based on probability distribution which is another type of fine registration [9].

In general, common coarse registration methods are based on local geometric features description, which includes the extraction of geometric features and the determination of correspondences. Many approaches of extracting the feature point have been widely reported. Li proposed an improved Harris algorithm by combining the discrete curvature and the normal vector to extract feature [10]. The SIFT operator can reduce the influence of scale change on key point search, but its computation is complex [11, 12]. In the paper [13], a registration method combining with color moment information improves the registration accuracy. In the literature [14], the future points are obtained via 3D Difference of Gaussians over geometric scalar values of the points which ensures obtaining salient features. Prakhya S M calculated the HoNo (Histogram of Normal Orientations) at every point and detected the key point by evaluating the properties of both the HoNo and the neighborhood covariance matrix [15]. The point feature histogram (PFH) algorithm and the fast point feature histogram (FPFH) algorithm are popular algorithms of feature description [16–18], which generate a feature histogram for each point based on feature information. Prakhya S M et al. applied a binary quantization method on a state-of-the-art 3D feature descriptor [19], SHOT [20], and created a new binary 3D feature descriptor, B-SHOT. Kleppe A L introduced a descriptor of key point using conformal geometric algebra [21]. Instead of feature descriptor’s calculating and feature matching, the 4-Points Congruent Sets (4PCS) and semantic-key point based 4PCS (SK-4PCS) determine the corresponding four-point base sets by exploiting the rule of intersection ratios [22, 23]. Mellado et al. improved 4PCS and proposed SUPER 4PCS and speedups the registration process [24]. Another idea of coarse registration is Sample Consensus algorithm. For example, Ye et al. used Random Sample Consensus (RANSAC) algorithm to eliminate the wrong matches [25]. In the literature [26], during coarse registration stage, Random Sample Consensus (RANSAC) algorithm is used to obtain the transformation between two 3D point clouds. The Normal distributions transform (NDT) algorithm is used to solve 2-D registration problem in the paper [27]. And Magnusson applied it in a 3-D space [28]. The NDT algorithm uses statistical probability method to determine the corresponding point pairs according to the normal distribution. Hong et al. proposed a probabilistic normal distributions transform (PNDT) representation which improves the accuracy of point cloud registration by using the probabilities of point samples [29]. Huan Lei et al. present a robust global approach for point cloud registration from uniformly sampled points, based on eigenvalues and normals computed from multiple scales [30].

Different from the above methods, this paper presents a key point selection algorithm which has a better anti-noise and fast ability. The feature descriptor of key points is highly exclusive which is based on the geometric relationship between the neighborhood points and the center of gravity of the neighborhood. We validate our proposal by presenting a quantitative experimental comparison with state-of-the-art methods. Experimental results show that the proposed point cloud registration algorithm has faster calculation speed, higher registration accuracy, and better anti-noise performance.

The rest of the paper is structured as follows. In Section 2, We introduce the principle of the algorithm in detail. In Section 3, the effectiveness of the algorithm is shown by experiment. Section 4 concludes this paper.

## 2. Point cloud registration based on the feature histogram of local surface

**Fig 1:**
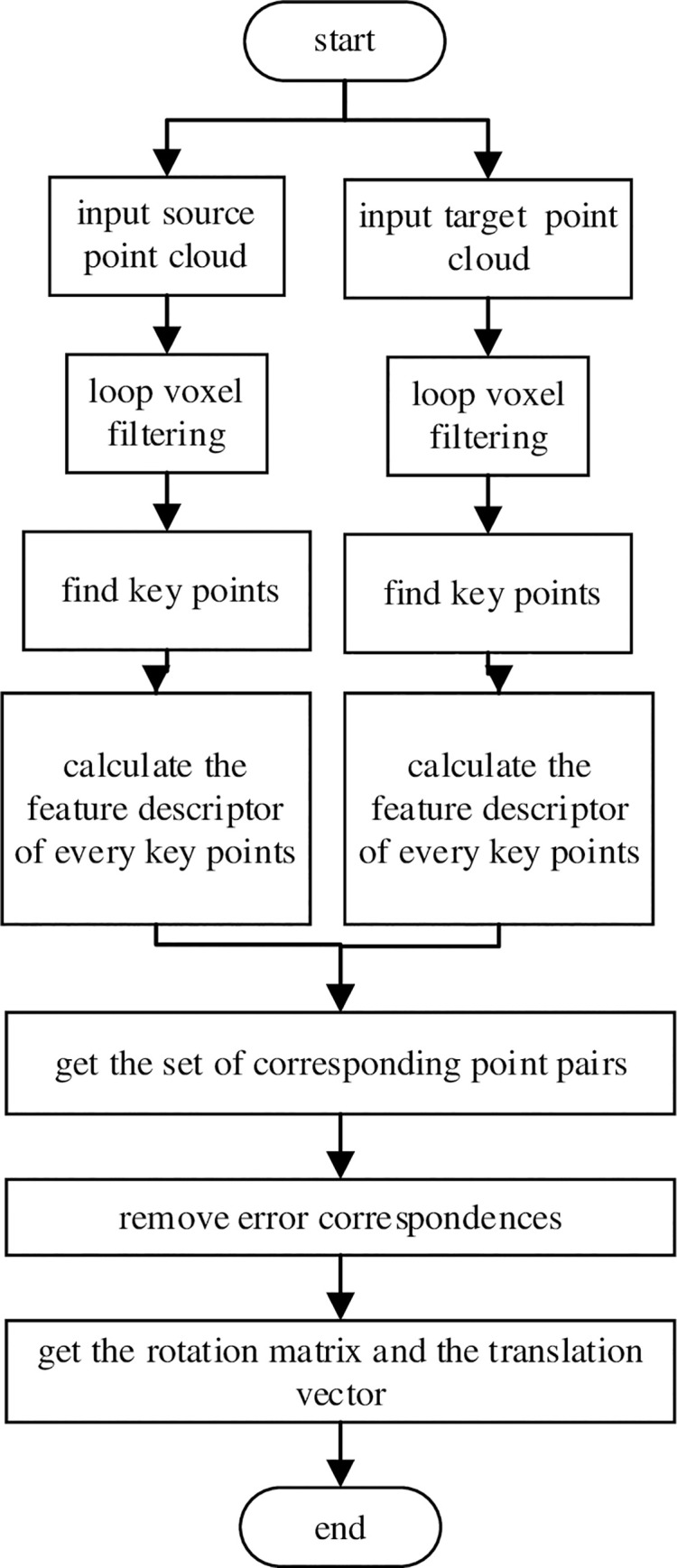
Flow chart of registration process.

The registration process in our method mainly includes Loop voxel filtering, Finding key points, The Feature Descriptor, Point cloud registration and other parts. The flow chart for the registration process is shown in Fig 1.

### 2.1. Loop voxel filtering

**Fig 2:**
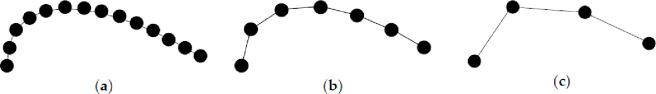
Point clouds of different densities. (a) The original dense point cloud; (b) the filtered point cloud; (c) the over-filtered point cloud.

The resolutions between different point clouds by using different acquisition equipment for different objects have a large difference, which leads that the multiple parameters should be set manually during the registration process. If the point clouds have too many points, the registration time will greatly increase. The point cloud filtering can deal with above problems. Compared to the filtered point cloud, original dense point cloud uses more points to describe the object surface. As shown in Fig 2, in order to describe the same surface, it requires 17 points in the original dense point cloud, but in filtered point only 7 points. Over-filtered point cloud cannot describe the surface correctly as shown in Fig 2(C).

The resolution of a point cloud is the average of the distances between each point and its nearest neighborhood point in the point cloud. The resolution describes the sparsity of point clouds. The greater the resolution, the sparser the point cloud. In order to achieve fast automatic registration, the point cloud resolution should to be calculated first:

$$s=\frac{\sum_{i=1}^{n}\|{p_{i}-p_{i}^{'}\|}_{2}}{n}$$ (1)

where ***p***_*i*_ is the i-th point in the point cloud, $p_{i}^{\prime }$ is its nearest neighbor point and *n* is the number of points in the point cloud.

In order to reduce point cloud size, voxel filtering will be used. The three-dimensional voxel grid is created in which each point is represented by the center of gravity of the grid. In this paper, an automatic voxel filtering on the point cloud is designed.

To improve registration efficiency, point clouds are filtered with uniform resolution of 1.0 mm. Automatic loop voxel filtering is adopted which calculates the maximum and minimum values of the x, y and z axis of the input point cloud, and establishes a three-dimensional bounding box according to these values and divides the bounding box into small cubes with the assigned voxel size, and represents all points in the small cube with the center of gravity of the small cube. In this way, multiple points inside the voxel are represented by one point, and the point cloud is reduced.

The filtering algorithm in this paper is implemented as follows:

1. Filter the original point cloud by using voxel filtering, and set the final voxel size, *s*_*target*_, to 1.0 mm.
2. Calculate the resolution of filtered point cloud, *s*_*now*_.
3. If 1.02* *s*_*now*_ is greater than *s*_*target*_, end the filtering process, else take voxel filtering on the filtered point cloud again, and the voxel size *s*_*loop*_ is determined by:
  $$s_{loop}=s_{target}+0.2\times (s_{target}-s_{now})$$ (2)
4. Go to step 2.

The filtered point cloud after above steps will be used as the initial point cloud for registration in the following sections, and its resolution is represented by *s*_*n*_ which will be used in the following sections also.

### 2.2. Finding key points

**Fig 3:**
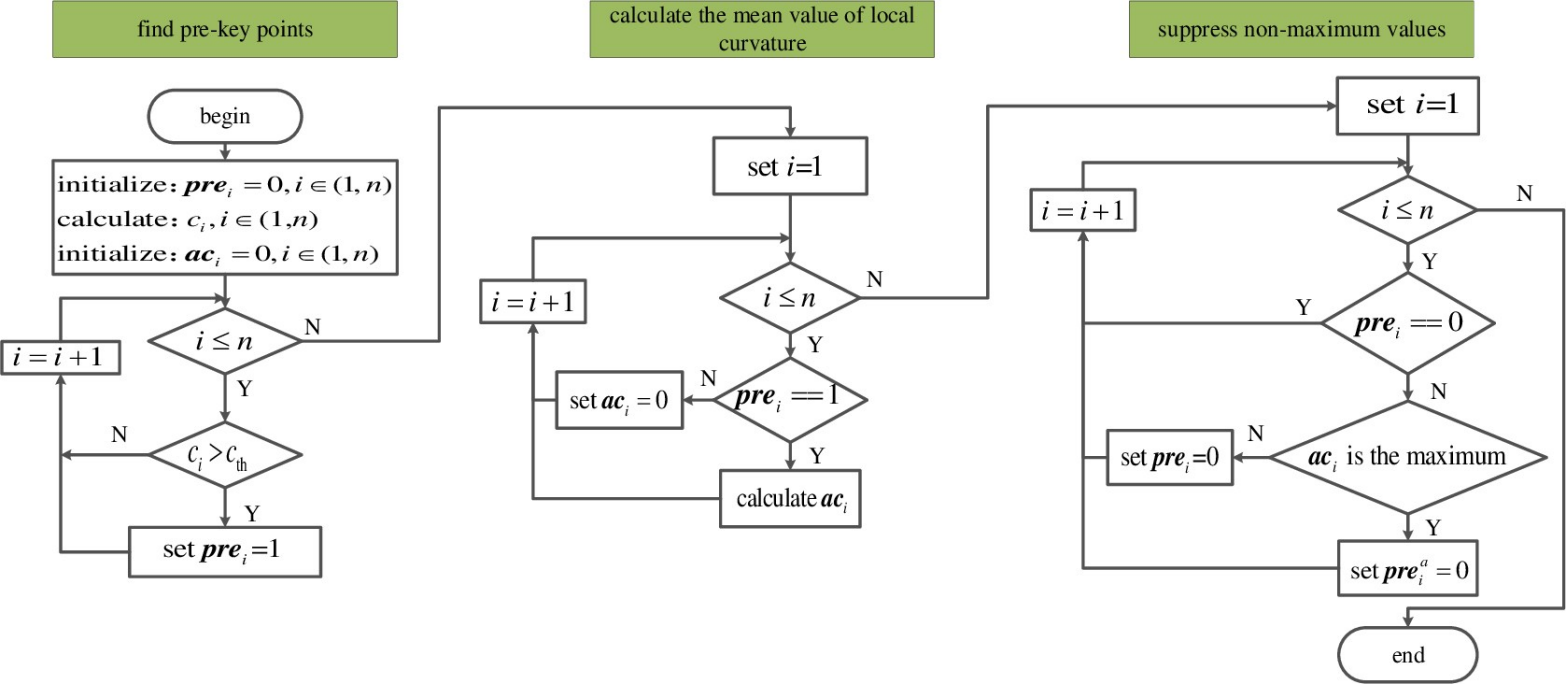
Key points finding algorithm.

After the point cloud filtering, the points are still redundant for the registration of point clouds. Most of the points locate at locations where local features are not apparent such as flat region. To improve the speed of registration, the key points are found for the registration. In order to find key points, classic algorithms based on a single point feature is sensitive to noise. In order to strengthen the resistance to noise, a finding algorithm of key point based on the biggest mean curvature of the pre-keypoint in its neighborhood is proposed. The algorithm proposed in this paper has better performance than the algorithm which only relies on single point’s curvature value. The key points finding algorithm is shown in Fig 3.

The key points are obtained based on point neighborhood. The neighborhood of a point *p*_*i*_ is defined as the set which includes all points within the sphere with center *p*_*i*_ and the radius *r*, where *r* = 5*s_*n*_. *s*_*n*_ is the current point cloud resolution. The covariance matrix *E* with dimension 3*3 and the eigenvalues *λ*_*1*_, *λ*_*2*_, *λ*_*3*_ based on the neighborhood of *p*_*i*_ are calculated:

$$E=\frac{1}{m}\sum_{a=1}^{m}\left(p_{i}^{a}-{\bar{p}}_{i}\right)\cdot {\left(p_{i}^{a}-{\bar{p}}_{i}\right)}^{T}$$ (3)

$$E\cdot \upsilon_{j}=\lambda \cdot \upsilon_{j},j\in \left\{1,2,3\right\}$$ (4)

where $p_{i}^{a}$, a ∈(1, *m*) is the *a*-th point in the Neighborhood of *p*_*i*_ and *m* is the number of points in the neighborhood of the point ***p***_*i*_. ${\bar{p}}_{i}$ is the centroid of the neighborhood of ***p***_*i*_. *λ*_*j*_ and ***ν***_*j*_ are the eigenvalue and eigenvector of the covariance matrix ***E***, correspondingly. *λ*_*1*_ is the smallest eigenvalue. The curvature can be estimated from the above eigenvalues. The curvature *c*_*i*_ of the point ***p***_*i*_ is obtained by the following formula:

$$c_{i}=\frac{\lambda_{1}}{\lambda_{1}+\lambda_{2}+\lambda_{3}}$$ (5)

To speed up searching key points, the points whose curvatures are greater than the threshold *c*_*th*_ are chosen as candidate key points. The threshold of curvatures is *c*_*th*_ = *c*_*max*_—(*c*_*max*_-*c*_*min*_)/3, where *c*_*max*_ and *c*_*min*_ are the maximum and minimal curvatures in the whole cloud points, respectively. An n-dimensional column vector ***pre*** is established to store the flags which indicate whether each point in the point cloud is a candidate key point. Where *n* is the number of points of the point cloud. The initial value of ***pre*** is an all-zero vector, i.e. *pre*_*i*_ = 0, *i*∈(1, *n*). It means that the *i*-th point is not a pre-key point. If the curvature of i-th point is greater than *c*_*th*_, *pre*_*i*_ is set to 1 and make it as a pre-key point.

We use the symbol *ac*_*i*_ to represent the mean value of curvature of all points in the neighborhood of the *i*^*th*^ point. The neighborhood radius is *r*. If the point ***p***_*i*_ is not a pre-key point, its curvature mean value is set to 0, *ac*_*i*_ = 0. If the point ***p***_*i*_ is a pre-key point, the mean curvature of its neighborhood *ac*_*i*_ would be calculated:

$$ac_{i}=\frac{\sum_{a=1}^{m}c_{i}^{a}}{m}$$ (6)

where $c_{i}^{a}$ is the curvature of the point $p_{i}^{a}$ and *m* is the number of neighborhood points. The neighborhood point is denoted by $p_{i}^{a}$.

In the process of determining whether the pre-key point has the largest mean value of the curvature, the point’s pre-key point flag *pre*_*i*_ is set to 0 when its curvature mean *ac*_*i*_ is less than its surrounding points’ value. It can reduce the number of pre-key points and accelerate the calculation of curvature mean. The mean value of the curvature of the pre-key point would be compared with those of its neighbor points. If the mean value of the curvature of the pre-key point is larger than all its neighbor points, the pre-key point flag would be set to 1 and the pre-key point flag of its neighbor points would be set to 0. After above procedure, the points whose pre-key point flag are still 1 are taken as the final key points *p*_*k*_.

**Fig 4:**
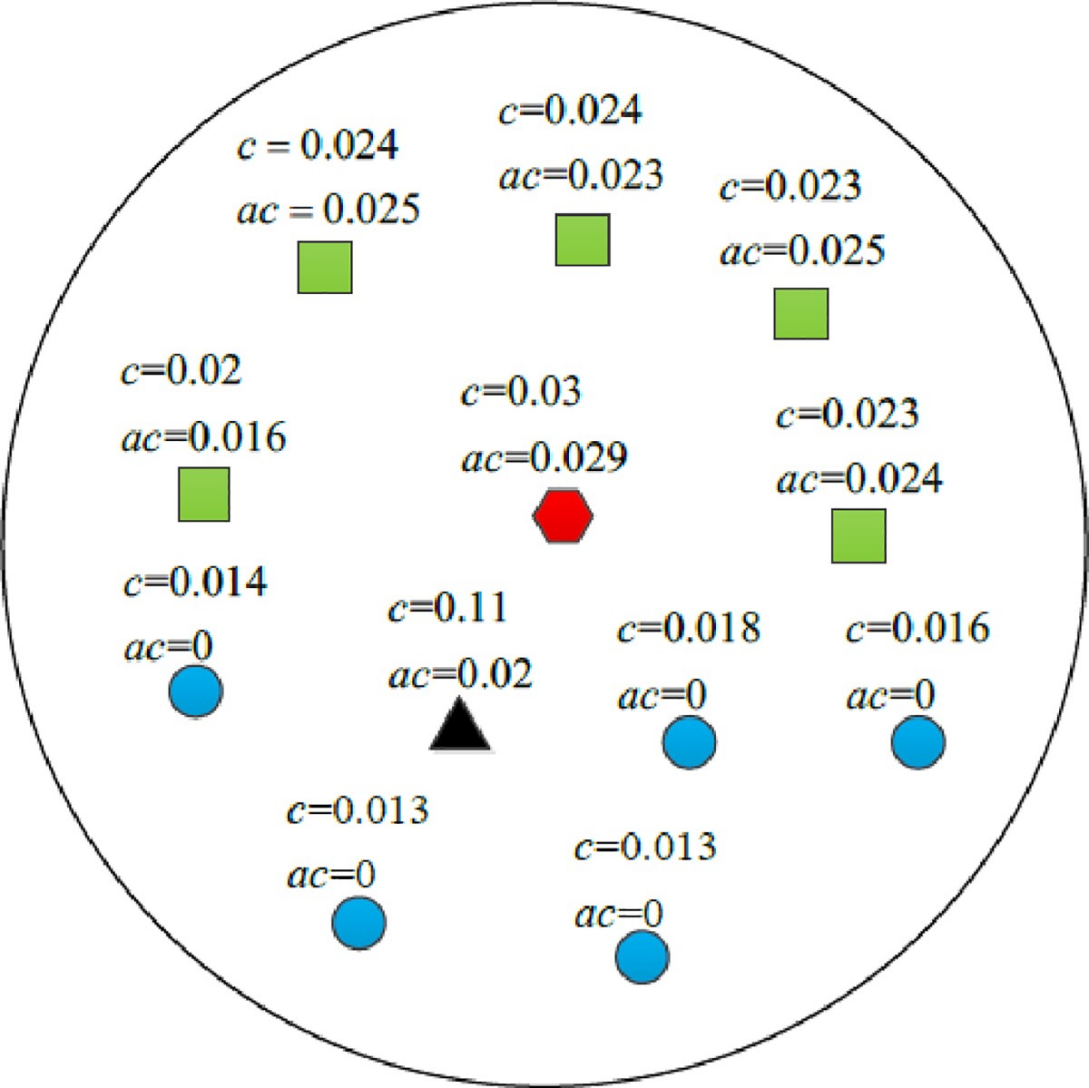
Anti-noise principle of finding algorithm of key points.

The anti-noise principle of the finding algorithm of key point is shown in Fig 4, where *c*_*th*_ = 0.02. The curvature of the circular points are less than 0.02, which indicates these points are normal. Since curvature of the square points are greater than 0.02, they are pre-key points. The curvature of the triangle point is abnormal, it is a noise point. The mean curvature of the neighborhood of the hexagonal point is the maximum in its neighborhood, which is the key point. Due to the abnormal curvature of the noise point, the noise point would be mistaken as a key point according to its curvature only. Selecting the key point according to the mean of the neighborhood curvature can improve the anti-noise ability of the key point search algorithm. Although the curvature of the noise point is large, the mean of the neighborhood curvature of the neighborhood points are increased, the red hexagonal point can still be correctly selected as the key point.

### 2.3. The feature descriptor

The classical feature descriptor depends on the relationship between the key point and its neighborhood points. Due to abnormal information such as the normal of the noise point, when noise is mistaken as a key point, feature descriptors cannot correctly describe the geometric features of key point based on its neighbor information. For this reason, we propose a feature descriptor in which the local surface histogram is calculated according to the distance between the neighbor points and the gravity center of neighborhood of the key point, as well as normal of points in the neighborhood.

**Fig 5:**
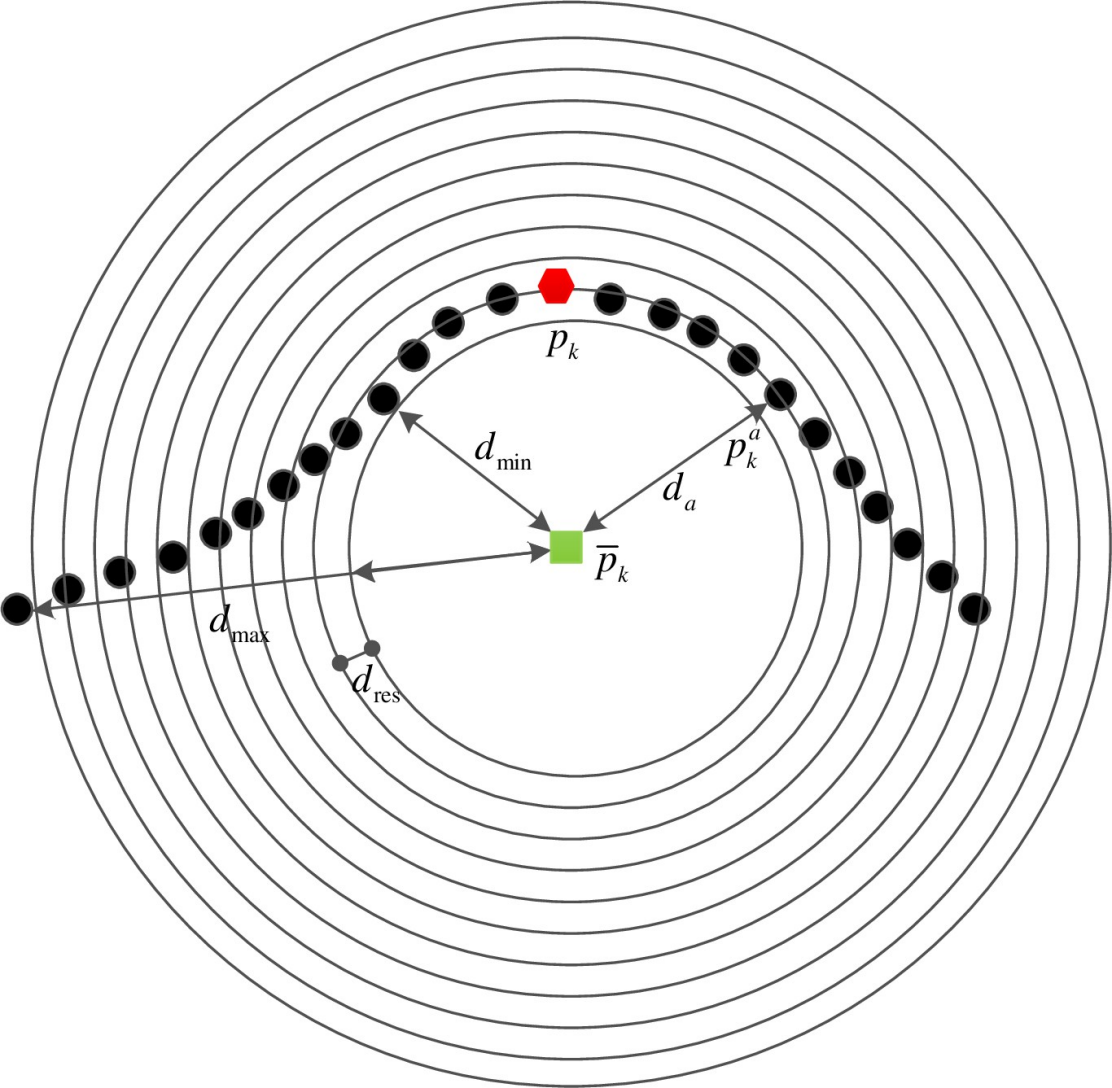
Grouping schematic of distances.

The radius of the neighborhood of the key point ***p***_*k*_ is denoted by *r*. The center of gravity ${\bar{p}}_{k}$ of the neighborhood is shown in Fig 5. The *d*_*a*_ is the distance from the neighborhood point $p_{k}^{a}$ to the center of gravity ${\bar{p}}_{k}$. The nearest distance is *d*_min_ and farthest distance is *d*_max_. The *d*_max_- *d*_min_ is divided into 10 parts.

The length of each part *d*_res_ is:

$$d_{res}=\frac{d_{\max}-d_{\min}}{10}$$ (7)

For the neighborhood point $p_{k}^{a}$, according to the distance *d*_*a*_, ${bin}_{d}^{a}$∈(1, 10) is computed as:

$$bin_{d}^{a}=\left\lceil \frac{d_{a}-d_{\min}}{d_{res}}\right\rceil$$ (8)

where ⌈⌉ means to round up to an integer.

**Fig 6:**
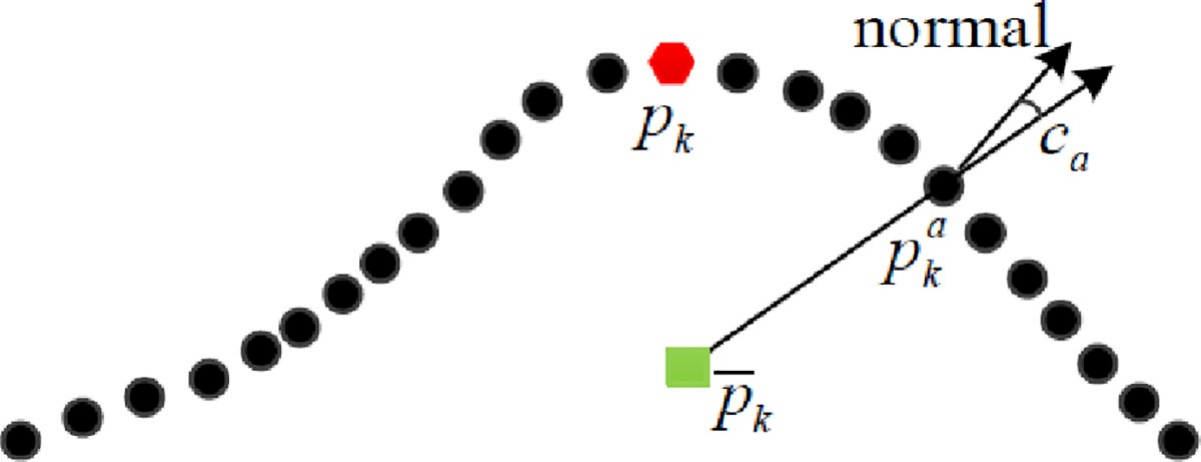
Schematic diagram of grouping cosine value.

The *c*_*a*_∈(-1,1) is cosine of the angle between the normal of the neighborhood point $p_{k}^{a}$ and the line from $p_{k}^{a}$ to the center of gravity ${\bar{p}}_{k}$, as shown in Fig 6. Where the hexagonal point ***p***_*k*_ is key point. The cosine value *c*_*a*_ is averagely divided into 12 parts.

Each part *c*_res_ is calculated as:

$$c_{res}=\frac{\left(+1\right)-\left(-1\right)}{12}$$ (9)

For the neighbor point $p_{k}^{a}$, its cosine value group number, ${bin}_{c}^{a}$∈(1,12) is calculated as

$$bin_{c}^{a}=\left\lceil \frac{c_{a}+1}{c_{res}}\right\rceil$$ (10)

The feature descriptor is calculated according to the geometric relationship between the center of gravity and neighbor points in the neighborhood of the key point. So the effect, when a noise is mistakenly chosen as a key point, can be reduced.

**Fig 7:**
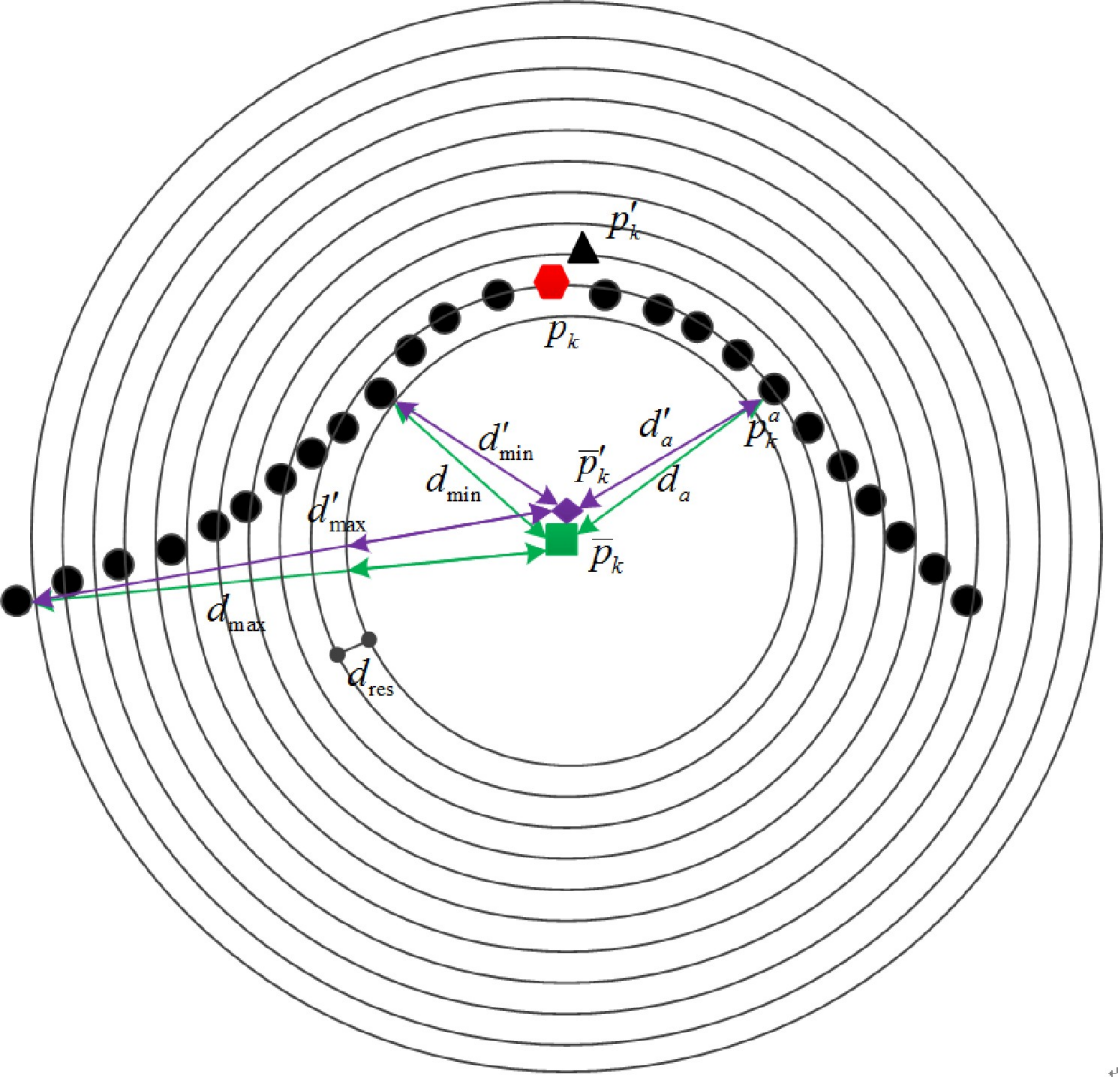
Anti-noise principle of distance grouping.

**Fig 8:**
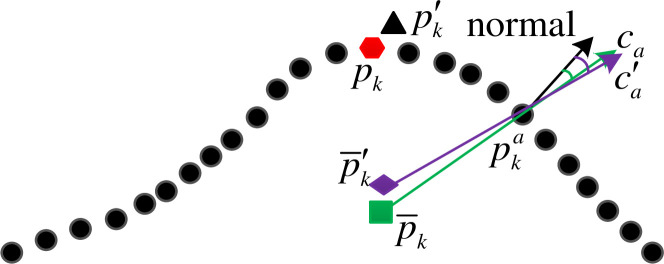
Anti-noise principle of cosine value grouping.

The anti-noise principle of the feature descriptors is shown in Figs 7 and 8, respectively. Where the hexagonal point ***p***_*k*_ is the true key point and the square point ${\bar{p}}_{k}$ is the gravity center of its neighborhood. The triangle point $p_{k}^{\prime }$ is a noise point and the diamond point ${\bar{p}}_{k}^{\prime }$ is the center of gravity of the neighborhood when the noise point $p_{k}^{\prime }$ is mistakenly chosen as a key point. As it can be seen from the figures, when the noise point is mistaken as a key point, the distinction between the two centers of gravity is small. At the same time, for the neighbor point $p_{k}^{a}$, the values (${bin}_{c}^{a},{bin}_{d}^{a}$) calculated by the wrong center of gravity also has small difference. The feature descriptor based on the noise point $p_{k}^{\prime }$ can still correctly describes the neighborhood.

A two-dimensional array *f*_12×10_ is used to store neighborhood information of the key point with 12×10 zeroes as initial values.

**Fig 9:**
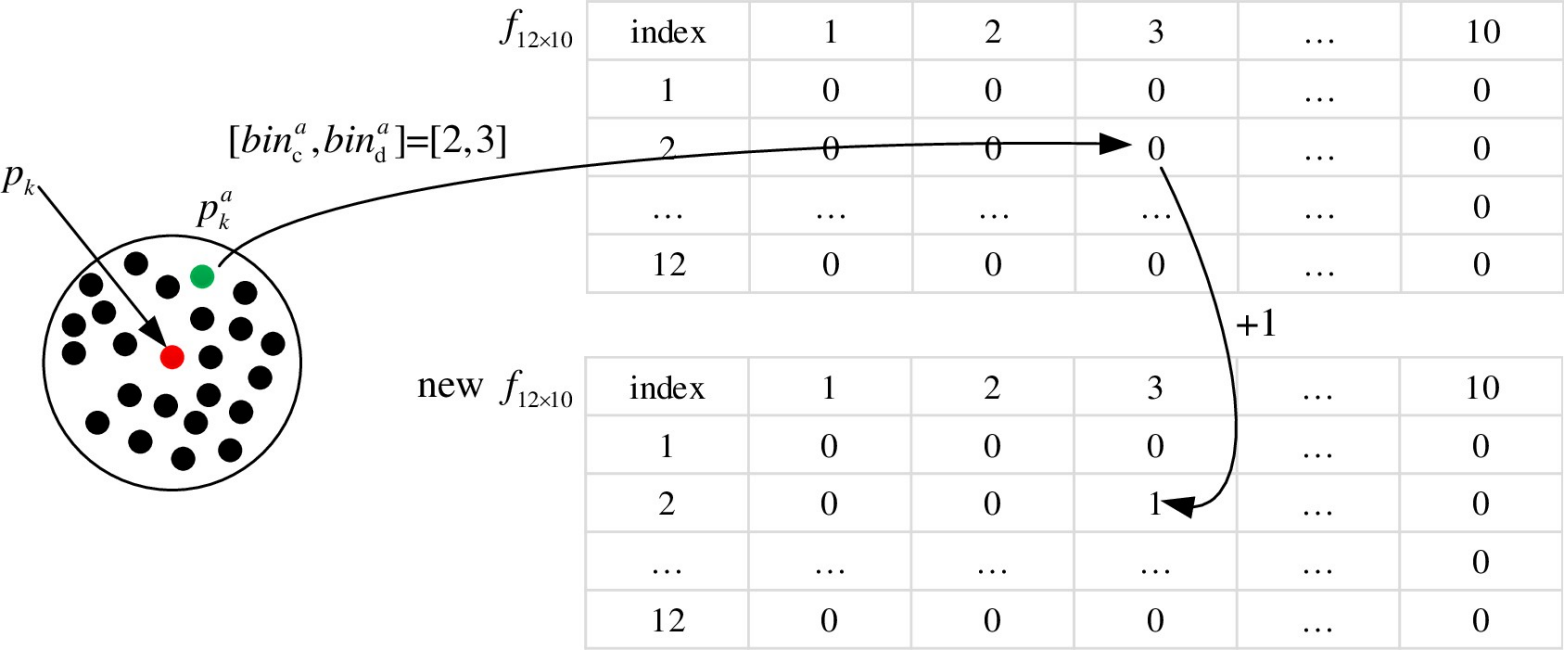
Calculation principle of the feature descriptor.

According to the values of *bin*_*c*_ and *bin*_*d*_ of neighbor point $p_{k}^{a}$, the value in the corresponding position of the 2D array *f*_12×10_ are added by one. As shown in Fig 9, the [*bin*_*c*_, *bin*_*d*_] of the neighbor point $p_{k}^{a}$ is [2, 3]. So the value in the position [2, 3] of the feature descriptor *f*_12×10_ of the key point ***p***_*k*_ are added by one.

To normalize the value in each position of the two-dimensional array *f*_12×10_, it is divide by the number of neighborhood points. After all points in the neighborhood of the key point are traversed, the two-dimensional array *f*_12×10_ is obtained and it is flattened to a column vector ***f*** of 120 rows. The column vector ***f*** is used as the feature descriptor for the key point *p*_*k*_.

### 2.4. Point cloud registration

The correspondences are determined based on the Euclidean distances of the descriptors of key points. The feature vector of key point $p_{k}^{s}$ of the source point cloud is represented by symbol $f_{k}^{s}$ and the feature vector of key point $p_{k}^{t}$ of the target point cloud is $f_{k}^{t}$:

$$f_{k}^{s}={\left[f_{k1}^{s}\;f_{k2}^{s}\;\cdots \;f_{k120}^{s}\right]}^{T}$$ (11)

$$f_{k}^{t}={\left[f_{k1}^{t}\;f_{k2}^{t}\;\cdots \;f_{k120}^{t}\right]}^{T}$$ (12)

The Euclidean distance between the feature vectors $f_{k}^{s}$ and $f_{k}^{t}$ is calculated by:

$$d\left(f_{k}^{s},f_{k}^{t}\right)=\sqrt{\sum_{j=1}^{120}{\left(f_{kj}^{s}-f_{kj}^{t}\right)}^{2}}$$ (13)

Since the feature descriptor has been normalized already, the average value of each dimension of the 120-dimensional feature descriptor, ***f***_avg_, is:

$$f_{avg}=\frac{1}{120}$$ (14)

When the difference between the key point feature descriptors of source point cloud and target point cloud is less than 0.5* ***f***_avg_, the mean square error *mse* satisfies:

$$mse<120\times {\left(0.5\times f_{avg}\right)}^{2}\approx 0.002$$ (15)

The kd-tree based on the descriptors of key points in source point cloud is generated. The closest key point in the target point cloud is searched in the generated kd-tree. If the mean square error of key point in the target point cloud is less than 0.002, the corresponding point pair will be added to the initial correspondence set *O*.

Because the feature descriptor describes the neighborhood information of the key point, if the neighborhoods of different key points are similar, some incorrect initial correspondences would be generated. In order to remove the incorrect correspondences, the neighborhood composite feature of the initial matching point pair is proposed in this paper.

**Fig 10:**
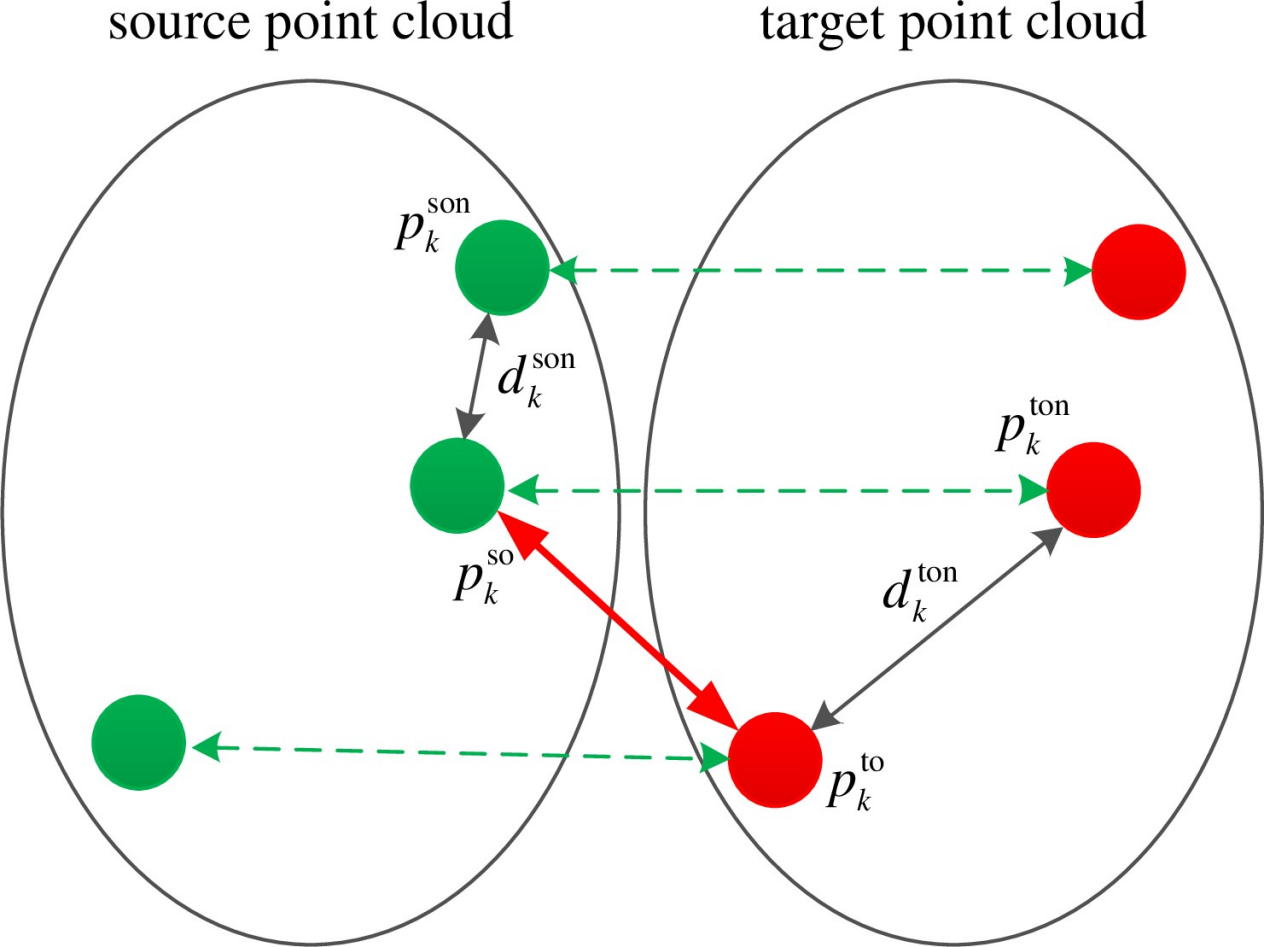
The removal principle of error correspondences.

Using the information of the Euclidean distance and feature descriptor of the nearest key point as a combined feature, the incorrect correspondence relationship is discarded according to the combined features. The nearest neighbor point $p_{k}^{son}$ for the key point $p_{k}^{so}$ in source point cloud is found, as shown in Fig 10. The $d_{k}^{son}$ is the distance between the point $p_{k}^{son}$ and $p_{k}^{so}$. The mean value of the descriptors of the point $p_{k}^{so}$ and the point $p_{k}^{son}$ is taken as the neighborhood composite feature $f_{k}^{so}$ of the point $p_{k}^{so}$. The nearest neighbor $p_{k}^{ton}$ for the key points $p_{k}^{to}$ in the target point cloud is found. The $d_{k}^{ton}$ is the distance between the points $p_{k}^{ton}$ and $p_{k}^{to}$. The mean value of the descriptors of the point $p_{k}^{to}$ and the point $p_{k}^{ton}$ is taken as the neighborhood composite feature $f_{k}^{to}$ of the point $p_{k}^{to}$. If the absolute value of the difference between $d_{k}^{son}$ and $d_{k}^{ton}$ is greater than 10 times resolution of source point cloud, the correspondence will be discarded. Otherwise, if the Euclidean distance between the vectors $f_{k}^{so}$ and $f_{k}^{to}$ is greater than 0.002, the correspondence is discarded. After above procedures, the final correspondence set is obtained. As shown in Fig 10, solid lines represent mismatches and dashed lines represent correct matches. Although the mean square error of feature descriptors of points $p_{k}^{so}$ and $p_{k}^{to}$ is small, the closest points distance $d_{k}^{son}$ and $d_{k}^{ton}$ are quite different. And the neighborhood composite features $f_{k}^{so}$ and $f_{k}^{to}$ are quite different. The incorrect correspondence can be effectively removed by comparing the closest point distance of the initial corresponding point pair with the neighborhood composite feature.

According to the final correspondence set, the rotation matrix and the translation vector between source point cloud and target point cloud are calculated by using the SVD algorithm and coarse registration is completed. Then the fine registration is finished by using ICP algorithm.

## 3. Experiment

**Fig 11:**
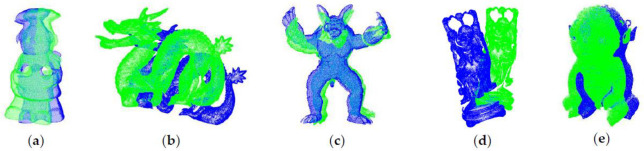
Initial positions of point clouds. (a) Cheff; (b) Dragon; (c) Armadill; (d) Happy; (e) Boy.

The initial positions of the point clouds are shown in Fig 11. The cheff_source, dragon_source, armadillo_source, happy_source and boy_source are source point clouds represented by green color. The cheff_target, dragon_target, armadillo_target, happy_target and boy_target are target point clouds represented by blue color.

The dense point clouds can be simplified to a specified resolution by using proposed algorithm in this paper. Table 1 shows the resolutions of point cloud during loop filtering where the specified resolution is 1mm. After several loops of voxel filtering, the point cloud can be automatically adjusted to the specified resolution. So the registration can deal with point clouds obtained by different scanners from different distance automatically, eliminating manual turning registration parameters which are based on point clouds with different sizes. After filtering, the resolution of source point cloud is almost the same as the target cloud with the error about 1.2%.

**Table 1:** Number of points in the process of loop voxel filtering.

Counts of iteration	dragon_source		dragon_target		armadillo_ source		armadillo_ target	
	point number	resolution (mm)	point number	resolution (mm)	point number	resolution (mm)	point number	resolution (mm)
0	100250	0.221882	43572	0.218354	172974	0.457438	83636	0.453643
1	59515	0.745332	26330	0.745497	54787	0.717815	27926	0.708482
2	45820	0.864262	20347	0.861433	39987	0.859273	20350	0.847701
3	40086	0.935212	17873	0.933344	33827	0.934583	17362	0.926101
4	36500	0.989653	16370	0.984749	30572	0.985521	15567	0.982849
Consuming time (ms)	218		94		203		109	

**Fig 12:**
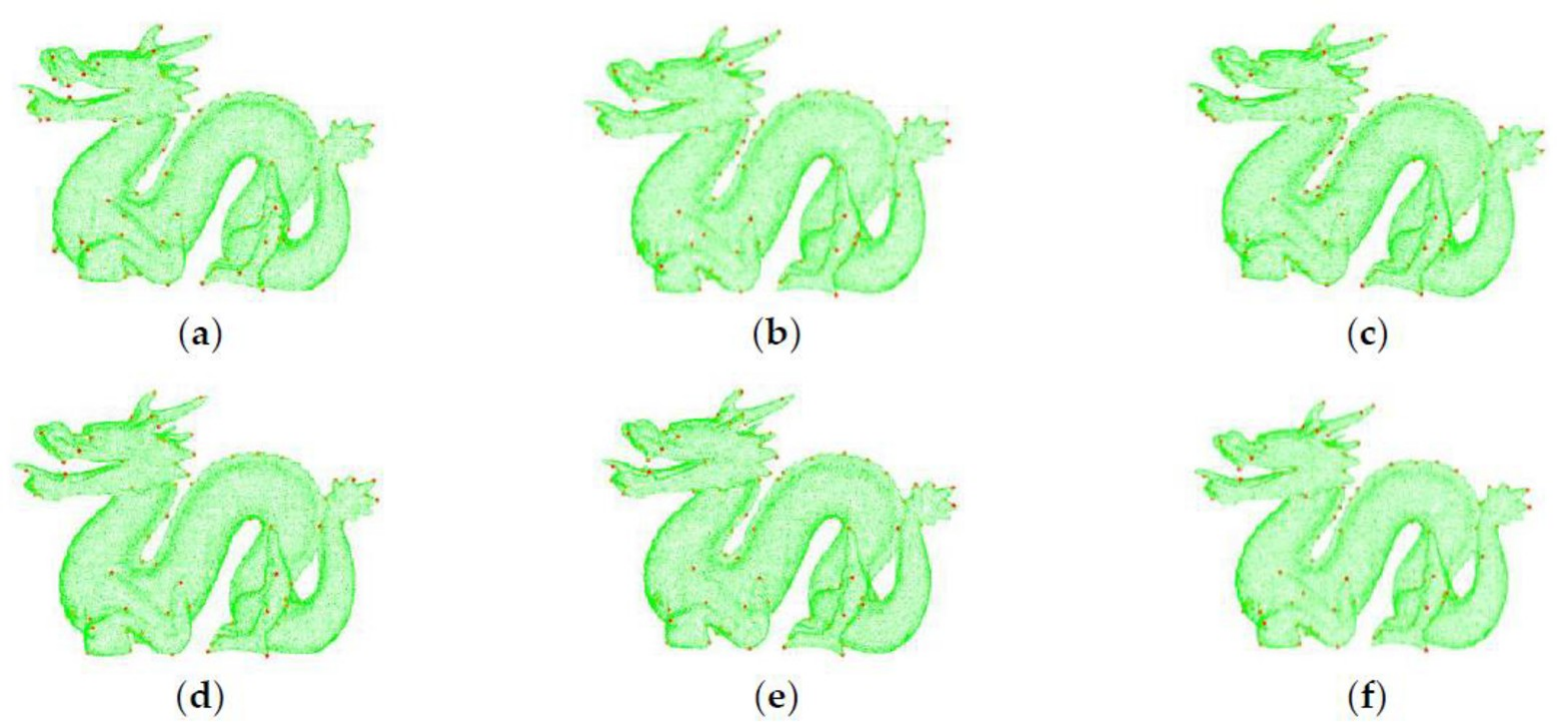
Key point distribution under noises. (a) *σ* = 0; (b) *σ* = 0.1; (c) *σ* = 0.2; (d) *σ* = 0.3; (e) *σ* = 0.4; (d) *σ* = 0.5.

The key point distributions of point cloud dragon_source under Gaussian noise with variance σ* s_n_ are shown in Fig 12. It can be seen that the key points obtained by the proposed algorithm based on the mean curvature of points in its neighborhood are more evenly distributed. The key points are located in the surface where the curvature changes greatly. After adding Gaussian noise, the key points are found in same place almost for different *σ* values that means the obtained key points are robust to noise.

The registration error is defined as the average distance between corresponding point pairs in the source point cloud and the target point cloud after registration. The smaller the registration error, the better the registration result. Table 2 shows that the registration algorithm proposed in this paper is fast and has high registration accuracy. When the noise is small, the registration accuracy of these registration algorithms is similar. With the increase of noise, the registration algorithm proposed in this paper is more accurate than PFH, FPFH and SHOT.

**Table 2:** Registration error under noises.

Noises	Registration error of cheff (mm)				Registration error of dragon (mm)			
	PFH	FPFH	SHOT	our method	PFH	FPFH	SHOT	our method
0	0.224574	0.273543	0.213564	0.192364	0.163234	0.152315	0.125342	0.134274
0.1	0.374634	0.425436	0.323743	0.204072	0.214734	0.225462	0.152362	0.147342
0.2	0.574965	0.567244	0.434583	0.321691	0.324374	0.337345	0.274245	0.252324
0.3	0.824546	0.765364	0.534296	0.384844	0.454536	0.473745	0.394554	0.355267
0.4	1.011268	0.950696	0.845454	0.515503	0.573454	0.601235	0.523512	0.512315
0.5	1.157674	1.157645	1.012543	0.634767	0.734572	0.953742	0.585596	0.561351
Time (ms)	6231	3257	2526	1423	4231	3021	2834	1637

Table 3 shows the calculation time of feature descriptors for different radiuses. As the radius of the neighborhood increases, the calculation time of PFH and FPFH grows faster, while the calculation time of SHOT and the algorithm proposed in this paper grows slower.

**Table 3:** The comparison of time of calculating feature descriptors.

Neighborhood radius (mm)	Time spent on calculating 300 feature descriptors (ms)			
	PFH	FPFH	SHOT	Our method
10	1346	970	186	124
20	14500	6406	250	164
30	76504	14312	410	228
40	240594	24906	532	376
50	556628	37532	906	504

When the same accuracy is achieved, the correspondence optimizing algorithm proposed in this paper has a shorter time than RANSAC, as shown in Table 4.

**Table 4:** Results of RANSAC and proposed algorithm for removing the error correspondence.

Algorithm	Consuming time (ms)		Registration error (mm)	
	cheff	dragon	cheff	dragon
Our method	332	316	0.141291	0.134632
RANSAC	1109	938	0.149769	0.132017

**Fig 13:**
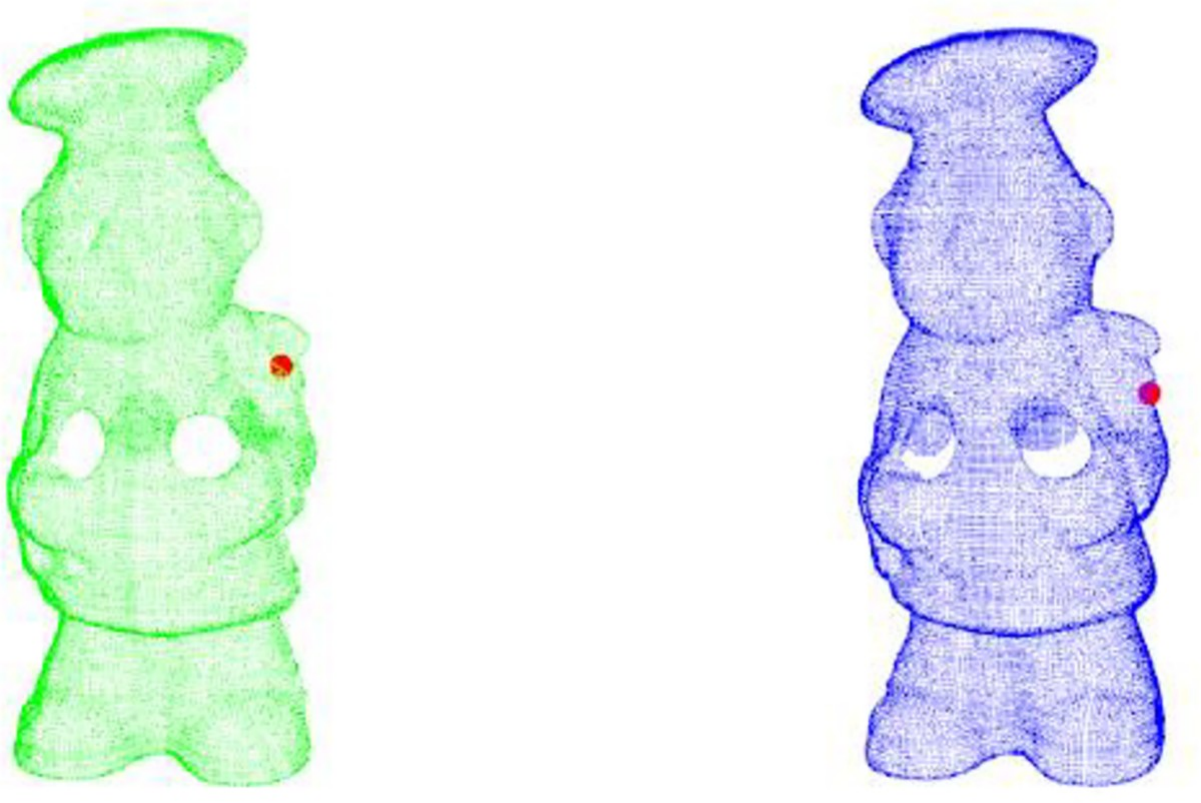
Wrong corresponding points in cheff.

**Fig 14:**
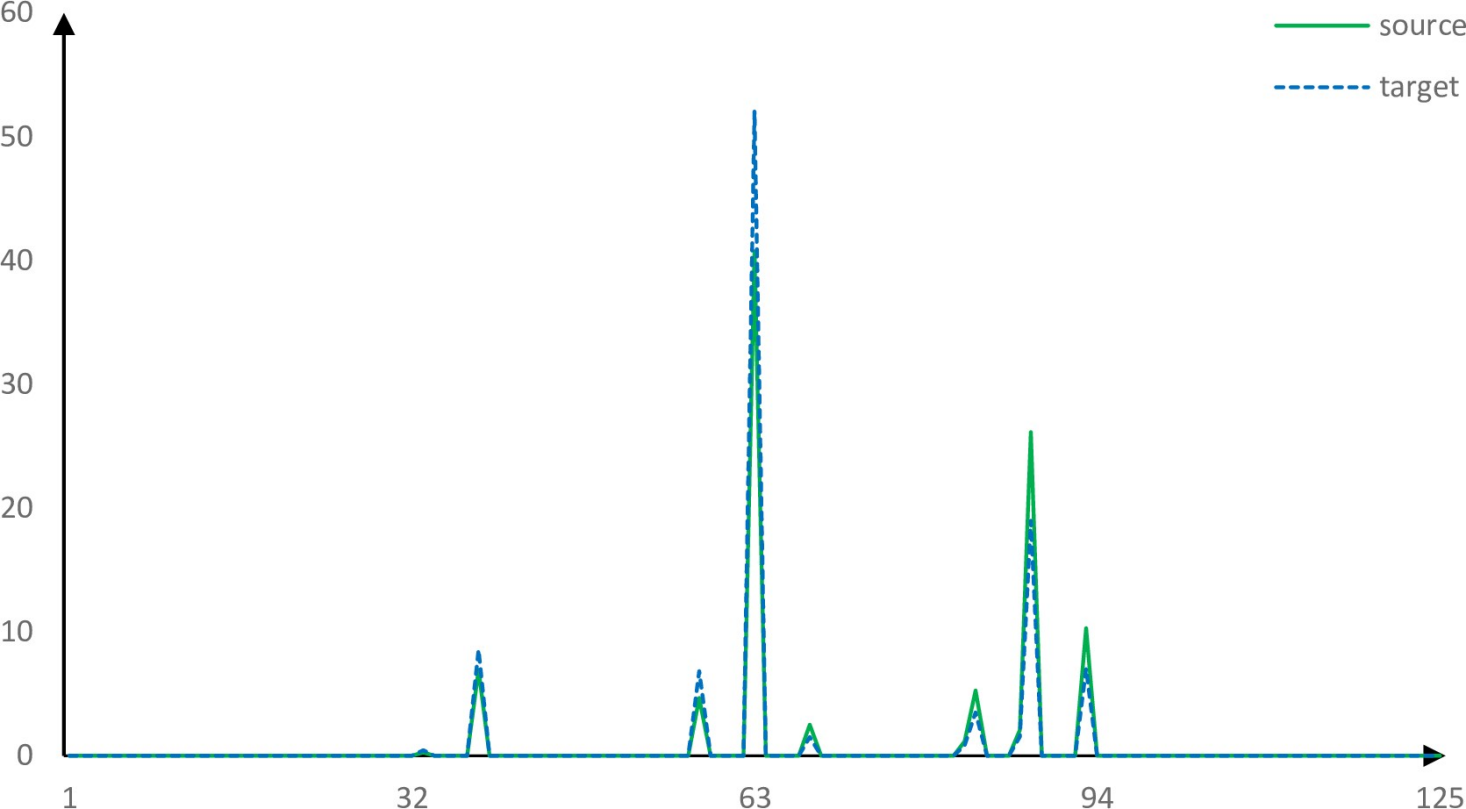
Corresponding points feature descriptors using PFH.

**Fig 15:**
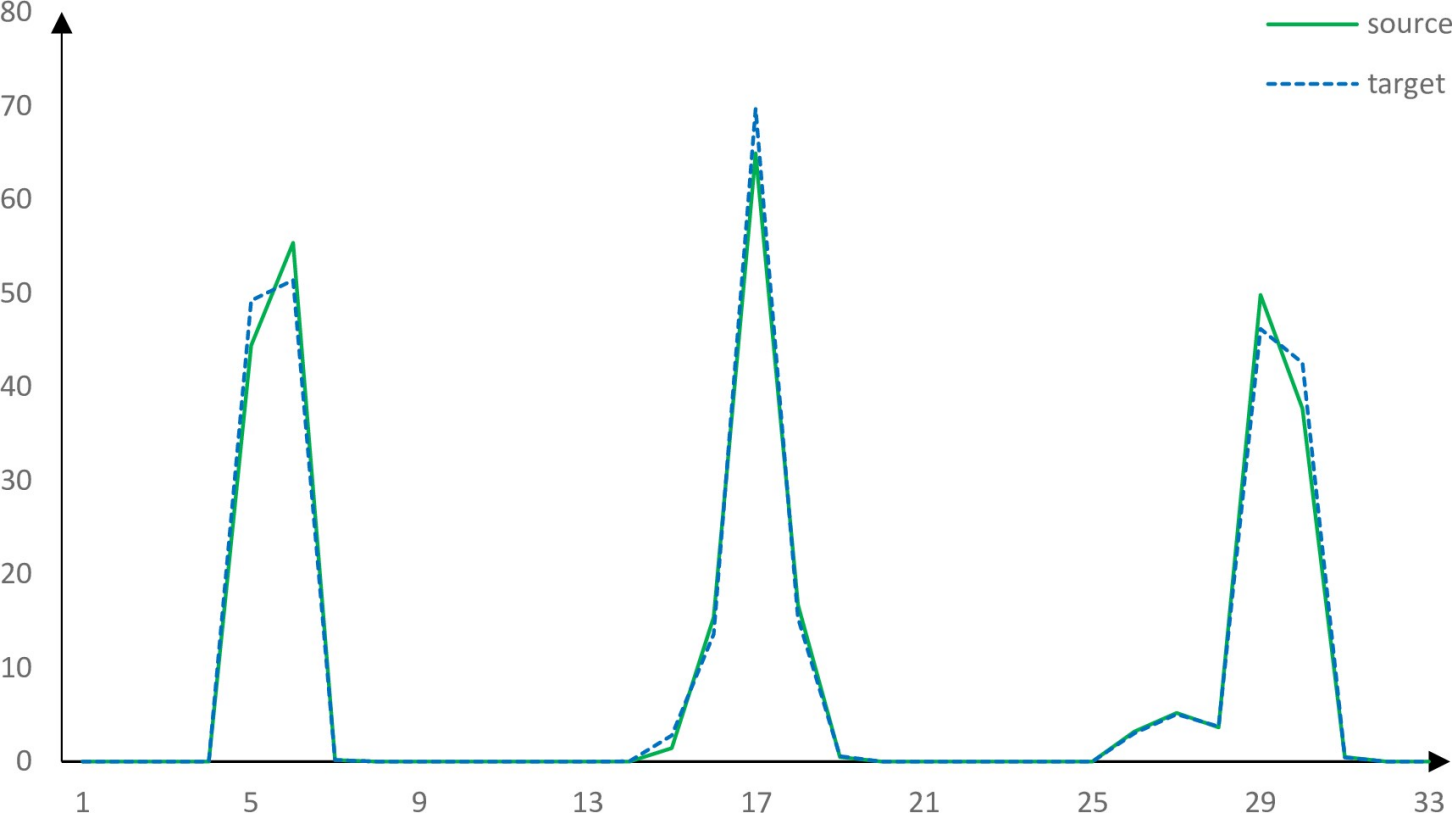
Corresponding points feature descriptors using FPFH.

**Fig 16:**
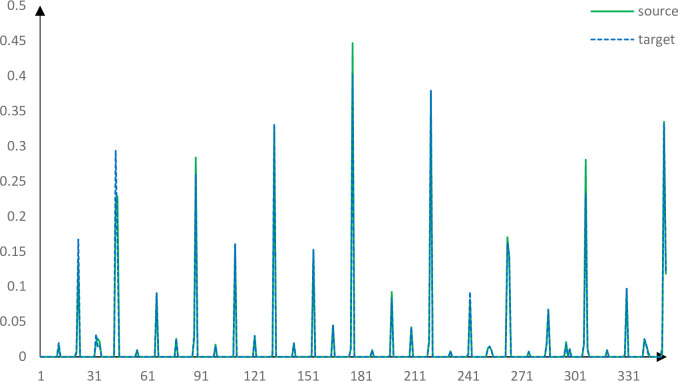
Corresponding points feature descriptors using SHOT.

**Fig 17:**
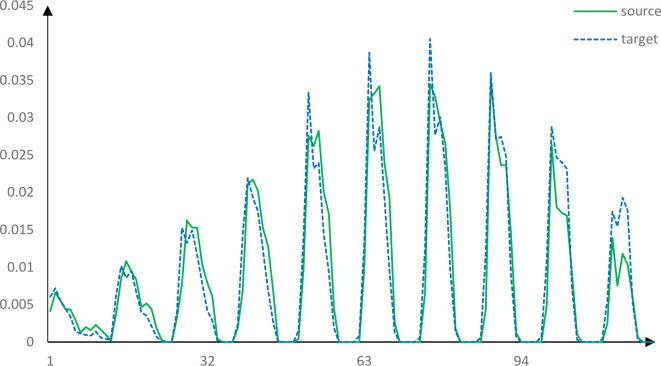
Corresponding points feature descriptors using our method.

The descriptors of two correct corresponding points obtained by using the PFH, FPFH, SHOT and our method are roughly similar. Figs 13–17 shows the wrong corresponding points and their feature descriptors obtained by these methods. The left side is the source point cloud, the right side is the target point cloud, and the big point is the corresponding point. Because the local features are similar, PFH, FPFH, and SHOT cannot distinguish the wrong corresponding points, but the feature descriptor proposed in this paper can still distinguish subtle differences. The feature descriptor proposed in this paper has better distinct ability with fewer dimensions.

**Fig 18:**
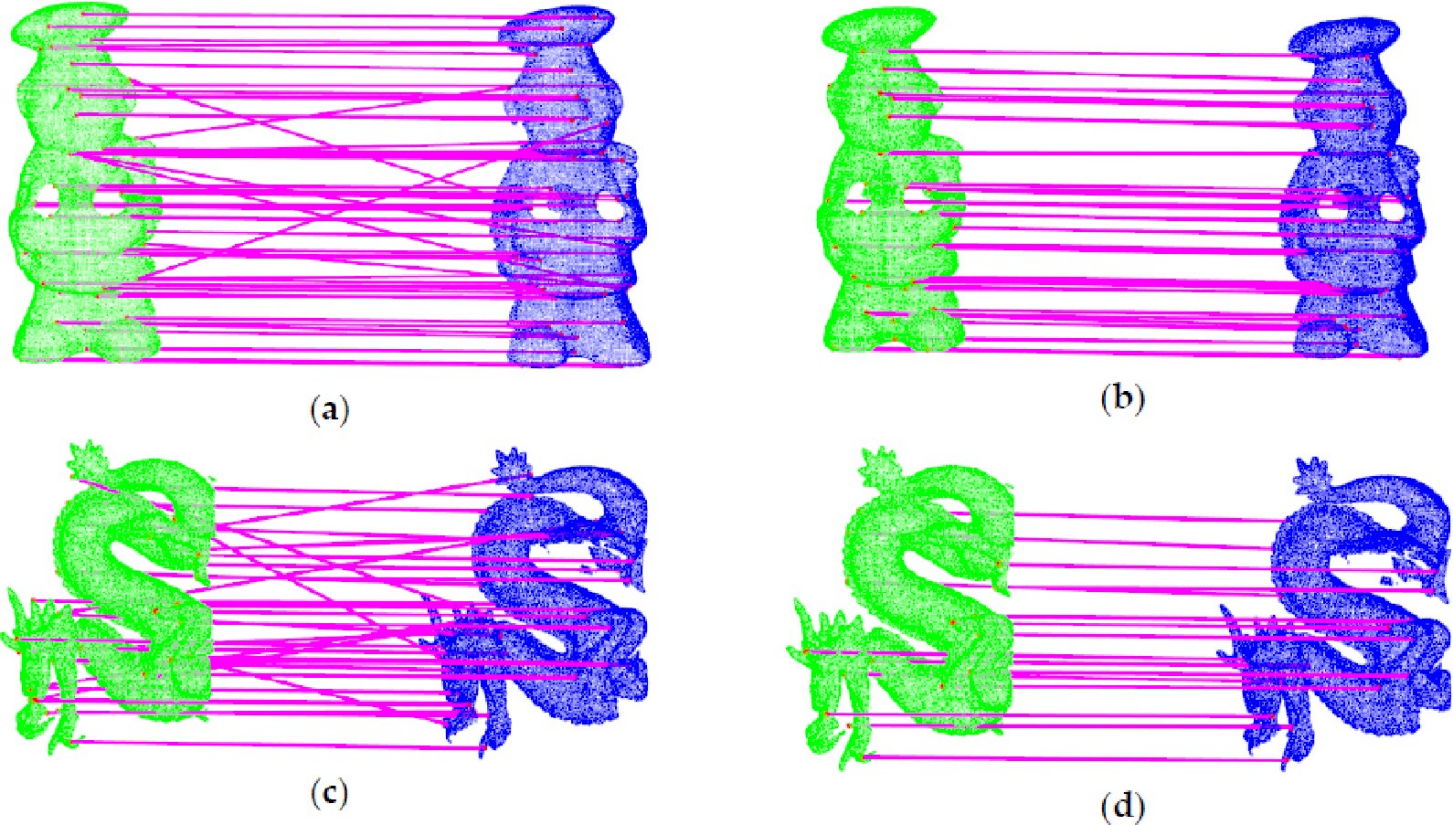
Comparison of the initial correspondences and correspondences after removing the errors. (a) Initial Correspondences Between a_s and a_t; (b) Final Correspondences Between a_s and a_t; (c) Initial Correspondences Between d_s and d_t; (d) Final Correspondences Between d_s and d_t.

As shown in Fig 18, there are many error correspondences through first matching. By using our method to remove the error correspondences, the final correspondences are basically correct.

The Table 5 is parameters and results of registration used by the algorithm proposed in this paper. The registration process is implemented automatically without human intervention.

**Table 5:** Parameters and results of registration.

Point cloud	cheff_s	cheff_t	dragon_s	dragon_t
Point number of clouds	89024	85794	187121	176930
Initial resolutions (mm)	0.59141	0.59243	0.22188	0.23245
Point number after filtering	35123	32634	31674	29457
Filtering time (ms)	145	132	215	228
Resolutions after filtering (mm)	0.983531	0.982354	0.987564	0.990124
Number of key points	226	215	263	274
The time of computing feature (ms)	156	163	175	179
Initial correspondences (pair)	142		157	
The time of determining initial correspondences (ms)	32		36	
Optimized correspondences (pair)	84		79	
The time of optimizing correspondences (ms)	146		147	
The time of SVD (ms)	344		323	
The time of ICP (ms)	425		585	
Registration error (mm)	0.190132		0.136213	

Table 6 shows the results of registration by tuning algorithm’s parameters manually. The filtering algorithm is voxel filtering. The voxel size is determined by multiple trials. The finding key point algorithm is based on uniform sampling. The sampling interval is 20 times the voxel size. The feature descriptor SHOT is adopted. The fault correspondences are removed by the RANSAC algorithm. Finally, ICP fine registration is performed.

**Table 6:** Parameters and results of registration.

Point cloud	cheff_s	cheff_t	dragon_s	dragon_t
Point numbers of clouds	89024	85794	187121	176930
Initial resolutions (mm)	0.59141	0.59243	0.22188	0.23245
Voxel size	1.45	1.45	1.35	1.35
Point numbers after filtering	24019	23508	22412	21536
Filtering time (ms)	47	35	74	71
Resolutions after filtering (mm)	1.04143	1.04626	0.951742	0.922374
Number of key points	160	155	184	186
The time of computing feature (ms)	231	215	205	195
Initial correspondences (pair)	196		161	
The time of determining initial correspondences (ms)	42		63	
Correspondences after removal process (pair)	74		84	
The time of removal process (ms)	1134		1237	
The time of ICP (ms)	863		910	
Registration error (mm)	0.210131		0.124432	

**Fig 19:**
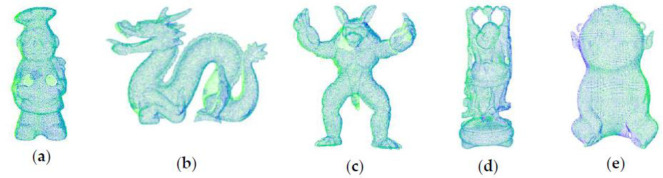
The results of point cloud registration. (a) Cheff; (b) Dragon; (c) Armadill; (d) Happy; (e) Boy.

Compared with the manually turning parameter algorithm, the filtering algorithm of this paper can automatically adjust the parameters according to the point cloud resolution. Despite the merely increased time, it is suitable for registration without manual intervention. Fig 19 shows the registration results of our algorithm with high registration accuracy.

**Fig 20:**
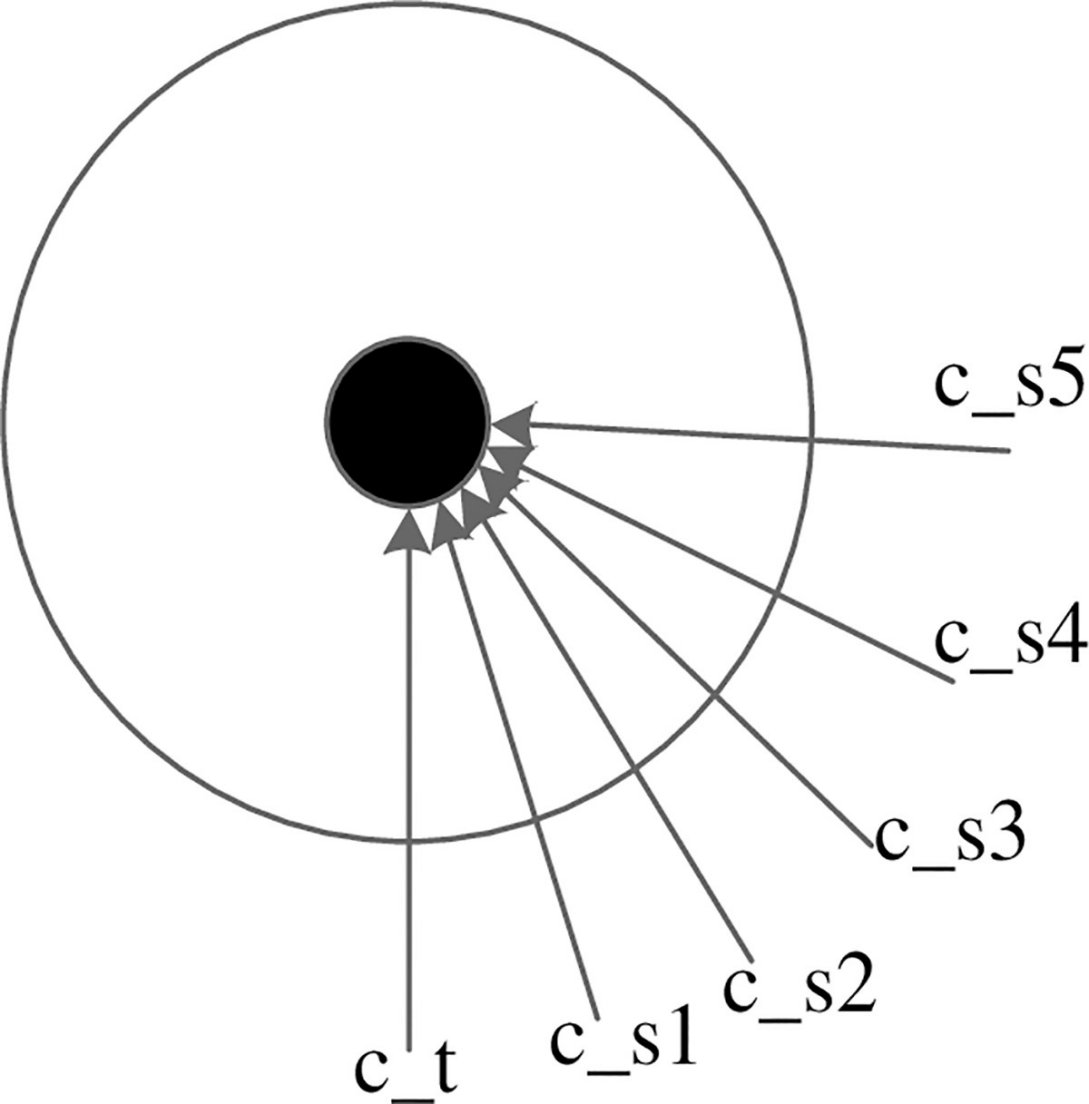
Six scan directions.

Fig 20 shows the six different scan directions to generate six point clouds of cheff. The six point clouds have different overlap rates. The overlap rate of two aligned point clouds is calculated as following. First search the closest point pairs in two aligned point clouds. When the distance between two closest points is less than five times the point cloud resolution, the points are viewed as overlapping point. The number of overlapping points is divided by the number of points in the point cloud as the overlap rate.

**Fig 21:**
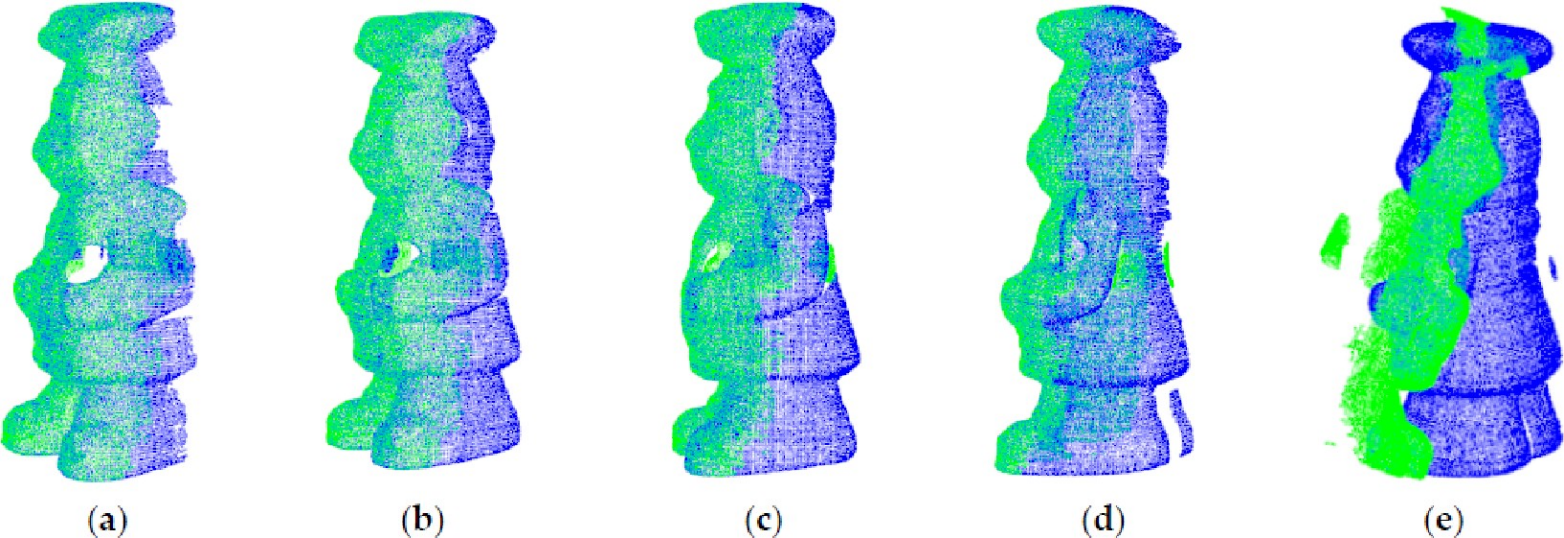
Registration results by using proposed algorithm. (a) c_s1 Registration Result; (b) c_s2 Registration Result; (c) c_s3 Registration Result; (d) c_s4 Registration Result; (e) c_s5 Registration Result.

**Fig 22:**
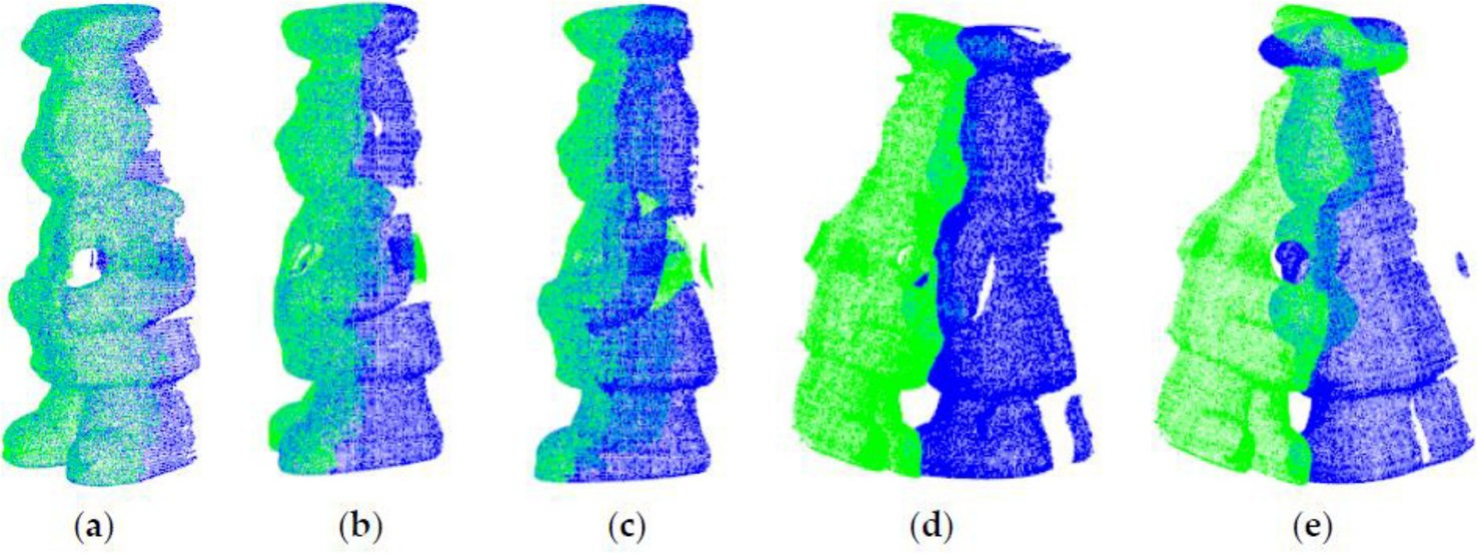
Registration results by using PFH algorithm. (a) c_s1 Registration Result; (b) c_s2 Registration Result; (c) c_s3 Registration Result; (d) c_s4 Registration Result; (e) c_s5 Registration Result.

As shown in Table 7, Figs 21 and 22, when the overlap rate is greater than or equal to 43.72%, our register algorithm has better accuracy. When the overlap rate is less than or equal to 37.52%, our algorithm fails to register. When the overlap rate is greater than or equal to 57.58%, the PFH algorithm can accurately register. When the overlap rate is less than or equal to 43.72%, the PFH algorithm fails to register. Due to the small number of key points in our registration algorithm, the difficulty of key point matching can be reduced and the registration effect can be maintained for the situation with a low overlap rate.

**Table 7:** Registration data of different overlap rate.

	c_t	c_s1	c_s2	c_s3	c_s4	c_s5
Point numbers of clouds	86884	90019	82949	76890	77467	81933
Rate of overlap (%)		89.74	71.55	57.58	43.72	37.52
Registration error of our paper		0.120578	0.143751	0.218378	0.242352	0.967535
Registration error of SHOT		0.132231	0.263515	0.326346	0.936345	0.977345

## 4. Conclusion

In this paper, the filter parameters are adaptively turned according to the resolution of the point cloud. A key point finding algorithm based on the mean value of the curvature of the neighborhood of the pre-keypoint is proposed. It did not adopt common key point finding algorithms which rely on single point curvature values and enhances robustness to noise, reduces the repetitiveness of key points at the same local region. In this paper, we proposed a computation method of feature descriptor based on distances and normal relationship between the center of gravity and each points in its neighborhood. Robustness to noise and uniqueness of the descriptor are improved. The wrong correspondences are removed effectively based on neighborhood combined feature of original matching point pair. It ensures accuracy of registration and reduces time of ICP. The proposed registration algorithm has good accuracy, computing efficiency and robustness to noise. It is suitable for automatic registration of point clouds with low overlapping rate and big noise.

## Supporting information

S1 File PCDATA. Point cloud data used in the paper.
(RAR)
